# Selection shapes malaria genomes and drives divergence between pathogens infecting hominids versus rodents

**DOI:** 10.1186/1471-2148-8-223

**Published:** 2008-07-30

**Authors:** Franck Prugnolle, Kate McGee, Jon Keebler, Philip Awadalla

**Affiliations:** 1 Laboratoire GEMI, UMR 2724 CNRS-IRD, 911 avenue Agropolis, BP 64501, 34394 Montpellier Cedex 5, France; 2 Department of Genetics, North Carolina State University, PO Box 7614, Raleigh 27659, USA; 3 Ste Justine Hospital Research Centre, Department of Pediatric, Faculty of Medicine, University of Montreal, Montreal H3T 1C5, Canada

## Abstract

**Background:**

Malaria kills more people worldwide than all inherited human genetic disorders combined. To characterize how the parasites causing this disease adapt to different host environments, we compared the evolutionary genomics of two distinct groups of malaria pathogens in order to identify critical properties associated with infection of different hosts: those parasites infecting hominids (*Plasmodium falciparum *and *P. reichenowi*) versus parasites infecting rodent hosts (*P. yoelii yoelii*, *P. berghei*, and *P. chabaudi*). Adaptation by the parasite to its host is likely highly critical to the evolution of these species.

**Results:**

Our comparative analysis suggests that patterns of molecular evolution in the hominid parasite lineage are generally similar to those of the rodent lineage but distinct in several aspects. The most rapidly evolving genes in both lineages are those involved in host-parasite interactions as well as those that show the lowest expression levels. However, we found that, similar to their respective mammal host lineages, parasite genomes infecting hominids are generally less constrained, evolving at faster rates, and accumulating more deleterious mutations than those infecting murids, which may reflect an historical lower effective size of the hominid lineage and relaxed host-driven selective pressures.

**Conclusion:**

Our study highlights for the first time the differences in trends and rates of evolution in *Plasmodium *lineages infecting different hosts and emphasizes the potential importance of the variation in effective size between lineages to explain variation in selective constraints among genomes.

## Background

A number of useful evolutionary parameters can be estimated from between species comparisons of genome-wide divergence patterns: the magnitude of positive and negative (purifying) selection, variation in selection across different lineages, chromosomes, gene families and individual genes as well as the number of genes involved in the speciation process and adaptation to new environments.

Comparative approaches have revealed that genes involved in immunity or in host defenses tend to exhibit the highest rate of evolution in the genome of different species. The arms race between hosts and parasites is generally invoked to explain this rapid evolution of genes involved in immune defense [1]. Pathogens continuously evolve to escape the defense of the host and the host, in turn, responds by modifying its defense. At the molecular level, this cycle of environmental changes means that new mutations are continuously tested and fixed by selection if adaptive, which translates into higher rates of molecular evolution in genes controlling immunity in the hosts.

While accelerated evolution of genes involved in immunity is common in mammals, the relative rate of evolution of those genes may vary from one phylogenetic lineage to another. This is the case for hominids (human and chimpanzee) compared to rodents (mouse and rats) where genes involved in immune defense show an accelerated rate of evolution in murids compared to hominids, suggesting that the immune system of murids has undergone more extensive specific innovations [2].

Hosts belonging to different lineages can therefore represent different environments for parasites to adapt. How do parasites preferentially infecting different host lineages respond to these different environments? Do parasites infecting different host lineages show lineage specific rates of evolution?

*Plasmodium *is a practical case study for genome evolution of parasites specifically infecting different host lineages. The genomes of two parasite species infecting only hominids (*Plasmodium falciparum *[3], *P. reichenowi *[4]) and three species preferentially infecting rodent hosts (*P. yoelii yoelii*, *P. chabaudi *and *P. berghei*) [5] are now partially or completely sequenced. Although these two groups of species might be subject to similar selective pressures acting either on the genome as a whole or on genes with similar function across species, some aspects of their genomes, such as genes associated with evading host immunity, may evolve in a unique manner.

**Figure 1:**
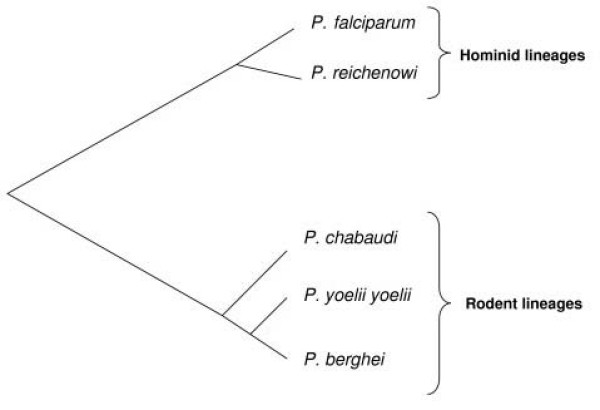
**Schematic representation of the phylogenetic relationship between hominid and rodent *Plasmodiu*m lineages (adapted from**[27]**).**

In this paper we systematically analyze and compare the rate of evolution of protein-coding genes in the parasites infecting hominids (hereafter called the hominid parasite lineage) and in those infecting rodents (rodent or murid parasite lineage) (Figure 1). We explore and compare the adaptive rate of evolution of genes in both groups based on their function and timing or level of expression, factors that may explain variation in the rate of evolution among different genes between the different lineages.

As for their two mammal host lineages (i.e. hominids and rodents) [2], our study reveals, in particular, that the evolution of the hominid lineage parasite genomes was less constrained than the evolution of those parasites infecting the murid lineage, which likely reflects a lower effective population size in hominid parasites (specifically *P. falciparum*).

## Results and Discussion

By taking the complete set of coding genes of *P. falciparum *and aligning it to a partial genome shotgun of *P. reichenowi *(covering approximately 1/3 of the entire genome) available in PlasmoDB, we identified a set of 843 pairs of genes with unambiguous orthology for which it was possible to generate high quality sequence alignments covering virtually the entire coding region (see Methods and Additional file 1). The same procedure retrieved 3060 triplets of orthologues for rodent malaria parasites *P. yoelii yoelii*, *P. berghei *and *P. chabaudi*, distributed throughout these genomes (Additional file 2).

### Average rates of evolution: evolutionary constraints and selection on amino-acid sites within the hominid and murid lineages

Analyzing estimates of ω (*dN/dS*) in both hominid (ω_Hominids_) and rodent (ω_rodents_) parasite species independently made it possible to study how evolutionary constraints and selection vary across both clades. In the hominid lineage, the average ω_Hominids _was estimated at 0.21 (Table 1), which is in congruence with previous estimates [4]. This excludes four genes with estimates of ω higher than 500 that had very low observed *dS *estimates (including these 4 genes, the average was ~4.68) and five genes with an undefined ratio (*dS *= 0). Among the 843 pairs of orthologues analyzed between *P. falciparum *and *P. reichenowi*, only ten pairs displayed a higher ratio than 1 but none were statistically significant (*p-values *less than 0.05 as determined by a likelihood ratio test). Most genes were conserved with ω significantly lower than one. A total of 719 genes, 85% of the genes analyzed, were in fact determined to be under purifying selection with ω significantly less than one.

**Table 1:** Evolutionary rates in hominid and rodent's *Plasmodium *lineage

	*Hominid lineage*	*Rodent (murid) lineage*
*dN*	0.012 ± 0.00044	0.026 ± 0.00041
*dS*	0.057 ± 0.0013	0.20 ± 0.0027
*dN/dS (ω)*	0.21 ± 0.0068	0.13 ± 0.0023

Maximum likelihood estimates of the rates of evolution (± standard error) in protein coding genes for hominid (*P. falciparum – P. rechenowi*) and rodent (*P. yoelii yoelii *– *P. berghei *– *P. chabaudi*) lineages. The difference between *dN/dS *in hominid and rodent lineages is significant. Note that the ratio of the means is not equivalent to the mean of the ratios.

The average ω _Murids _for the 3060 genes analyzed in *P. yoelii yoelii, P. chabaudi and P. berghei*, was estimated as 0.13 (Table 1), significantly lower than in parasites infecting hominids (Wilcoxon test over all genes, *p-value *< 10^-4^). Only 4 genes displayed a ratio greater than 1, but none were significant. In fact, more than 98% of the genes displayed a ratio significantly lower than 1.

Several observations suggest that the difference observed between the hominid and the rodent lineage is not due to the number of species aligned in each lineage nor their phylogenetic distances. First, the ω obtained between any pair of rodent parasite species, are similar, and lower than the estimate obtained for *P. falciparum *and *P. reichenowi *(data from [5]: *P. yoelii yoelii *and *P. chabaudi*: ω = 0.11, *P. yoelii yoelii *and *P. berghei*: ω = 0.16 and *P. chabaudi *and *P. berghei*: ω = 0.13). Second, the marked difference between hominid and rodent malaria parasites still held when comparisons between lineages are made in a paired way using only those genes from hominid and rodent lineages of known orthology (orthology between *P. falciparum and P. yoelii yoelii *was retrieved from [6]; Wilcoxon rank sum test; n (genes) = 263; *p-value *= 0.002). Therefore, there is an excess of about 25% of amino-acid altering substitutions, relative to synonymous substitutions, in the hominid lineage compared to the rodent lineage.

Interestingly, the host lineages of these two groups of parasites show similar differences in their rate of molecular evolution. Hominids show an average ω of 0.20, over the whole genome, while murids show an average ratio close to 0.13 [2,7]. As in the case of *Plasmodium *parasites, the difference between both estimates is highly significant. This difference in the rate of molecular evolution among the host lineages could be the result of two different processes: 1) a relaxation in the selective constraints acting on the amino-acid sequence in the host hominid lineage, relative to the murid lineage, subsequent to a reduction in their effective population size or 2) an acceleration in the global rate of adaptive evolution in hominids. Genetic evidence favors the first hypothesis [8,2,9]. Does the same explanation hold for parasites?

Comparing genetic variation at synonymous and non-synonymous sites within (polymorphism) and between species (divergence) can help distinguish between the two hypotheses above [10]. When most variation is neutral, the ratio of the number of nonsynonymous to synonymous polymorphisms observed within populations should be the same as the ratio of divergence between species [10]. Mu and collaborators [11] analyzed genome-wide polymorphism of 5 *P. falciparum *isolates distributed globally. Jeffares and collaborators also analyzed polymorphisms using three different African isolates [4]. In total, we obtained divergence and polymorphism data for 518 genes using Mu et al [11]'s data and 839 genes using Jeffares et al. [4]'s data. The ratio of the number of nonsynonymous to synonymous polymorphism was 2.3 for the first dataset and 2.7 for the second one. Comparatively, the ratio of divergence we observed among *P. falciparum-P. reichenowi *was only 1.1 and 1.3, respectively, thus indicating a 2-fold increase in the number of nonsynonymous polymorphisms. Although some amino-acid substitutions observed among species are likely adaptive, this observation supports selection being less efficient in removing segregating deleterious amino-acids in *P. falciparum*. A reduced effective size in hominid parasites compared to the murid lineage might be one explanation for the observed difference in ω. This scenario is supported by different studies suggesting the existence of an historical low effective population size in *P. falciparum*[12]. Unfortunately, no such population study exists for rodent malaria species. The evolution of both hominids and their malaria parasites appears to be less constrained than that of murids, which might reflect, for both the host and the parasite, a small historical effective size and an evolution mainly driven by slightly deleterious mutations [13].

### Variation in evolutionary rates across functionally different genes

We then asked whether specific groups of genes evolved differently from the rest of the genome. In particular, we searched for groups of genes that could have experienced an accelerated evolution in one lineage compared to the other, thus leading to a higher difference in their amino-acid substitution rate between lineages than expected given the difference observed across the genome.

**Figure 2:**
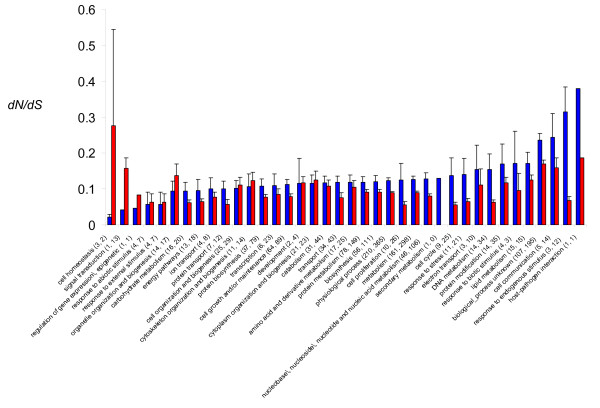
**Evolutionary rates (*dN/dS*) and Gene Ontology Processes in hominid (blue bars) and rodent (red bars) *Plasmodium *lineages**. The numbers between parentheses are the number of genes belonging to each group. The first number corresponds to the hominid lineage; the second corresponds to the rodent one.

To do so, we searched for variation in ω among different functional categories of genes. No functional annotation was directly available for the rodent lineage, so we classified rodent genes using their orthology with *P. falciparum *(see methods). Practically, because the amino-acid divergence was, on average, higher in the hominid lineage compared to the rodent lineage (see above section: Average rates of evolution), we sought categories that showed a significantly lower or higher difference than expected on average. To do so, we used the following Linear Model: ω ~ L + Ca + L*Ca + constant, where ω corresponds to the ratio computed for each gene, *L *to the lineage (Hominid or Rodent) and *Ca *to the category to which the gene belongs (the category of interest or the rest of the genome). A difference higher than the average ω for certain categories between the hominid and the rodent lineages should translate into a significant interaction (L*Ca). We considered only those categories that contained at least 5 genes in both hominid and rodent lineages. Overall, the ω ratio was correlated among categories between hominid and rodent lineages (*r*^2 ^= 0.34; *p-value *= 0.0019). We found no category of genes showing an accelerated evolution in one lineage compared to the other, relative to the rest of the genome (Fig. 2 and Table 2). Such a result can be interpreted in several ways. First, categories that could have been affected by such acceleration were not included in the analysis because of a lack of data. This could be the case, for instance, for categories of genes like those involved in host-parasite interactions like VAR genes which are not found in rodent parasite genomes. In our dataset, we had to exclude this category because of an insufficient number of genes belonging to it. Second, an evolutionary acceleration in the amino-acid substitution rate may not affect entire categories of genes but only some of them expressed, for instance, at only some particular stages of the parasite, thus rendering it more difficult to detect them.

**Table 2:** Go categories and relative divergence rates (*dN/dS*) in hominid and murid lineages

Go categories within "biological process"	*dN/dS (ω) * hominid	*dN/dS (ω) * murid	*p-value*
GO: Organelle organization and biogenesis	0.093797	0.136622	0.34
GO: Carbohydrate metabolism	0.094282	0.061524	0.17
GO: Energy pathways	0.095999	0.06409	0.25
GO: Protein transport	0.1000621	0.056915	0.10
GO: Cell organization and biogenesis	0.102521	0.11164	0.75
GO: Cytoskeleton organization and biogenesis	0.106537	0.123027	0.68
GO: Protein biosynthesis	0.10841	0.076408	0.092
GO: Transcription	0.1093033	0.085371	0.49
GO: Cell growth and/or maintenance	0.112558	0.078195	0.02
GO: Cytoplasm organization and biogenesis	0.115938	0.124335	0.80
GO: Catabolism	0.117659	0.107771	0.70
GO: Transport	0.118198	0.076005	0.059
GO: Amino acid and derivative metabolism	0.118466	0.105378	0.64
GO: Protein metabolism	0.118917	0.09077	0.06
GO: Biosynthesis	0.120925	0.09066	0.054
GO: Physiological process	0.12305	0.08881	0.0001
GO: Cell proliferation	0.125543	0.055601	0.03
GO: Metabolism	0.12623	0.0897	0.0001
GO: Nucleobase et al*.	0.128449	0.07981	0.01
GO: Cell cycle	0.137136	0.055177	0.01
GO: Response to stress	0.140927	0.064359	0.03
GO: DNA metabolism	0.154415	0.062976	0.0028
GO: Protein modification	0.170329	0.116675	0.21
GO: Lipid metabolism	0.171853	0.124668	0.17
GO: Biological_process unknown	0.2353	0.17011	0.001
GO: Cell communication	0.2439164	0.159463	0.17

*Nucleobase, nucleoside, nucleotide and nucleic acid metabolism.

Only those categories of biological processes with at least 5 genes in both lineages are listed. The *p-value *of the test comparing the average *dN/dS *(ω) ratio between hominid and murid lineages is given for each category (*p-value*). None of the category showed an accelerated evolution in hominid or rodent, given the average genome difference between lineages.

### Variation in evolutionary rates, timing and frequency of expression

We finally addressed how variation in evolutionary rates could evolve relative to the timing and the breadth of expression of the genes. Do genes expressed at a particular stage show lineage specific evolution?

**Figure 3:**
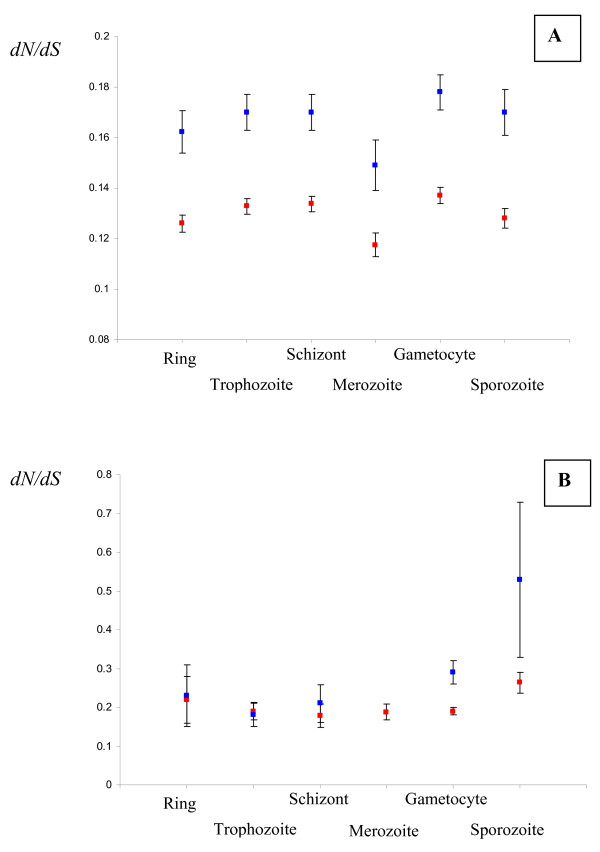
**A, B. Evolutionary rates (dN/dS) and timing of expression**. A. for all genes expressed at one stage (but that may also be expressed at another stage). B. for the genes that are only expressed at one particular stage. Blue squares: hominid lineage; Red squares: rodent lineage.

As shown in Fig. 3A, hominid and rodent genes show similar patterns of evolution relative to their timing of expression. While no significant difference was observed between categories of genes for hominids (*p-*value = 0.3), a difference was found between categories for rodents: (*p-*value = 0.013). For both lineages, genes that are expressed at the merozoite stage look the most constrained while those expressed at the gametocyte stage appear the least constrained. We did not find any group of genes showing an accelerated evolution in one lineage compared to the other relative to the rest of the genome.

The classification we used for Fig. 3A was nevertheless very broad. It included genes that were expressed at one particular stage but those same genes could also be expressed at other stages. Because this could preclude the detection of stage-specific evolution, we then re-analyzed our data keeping only the genes that were expressed at one stage. Doing so, we observed a significant difference in the rate of evolution among the different categories in the hominid lineage (*p-*value = 0.017) but no difference was observed in the rodent lineage (*p-*value = 0.59, Fig. 3B). We observed an overall difference between hominid and rodent parasites primarily due to the loci expressed at the sporozoite stage that showed an accelerated evolution in the hominid lineage compared to the rodent one (*p-*value = 0.018). This result suggests that genes only expressed at the sporozoite stage might be key genes in the infection process of the mammal host by malaria parasites and experienced higher adaptive evolution in the hominid lineage than in the rodent one.

**Figure 4:**
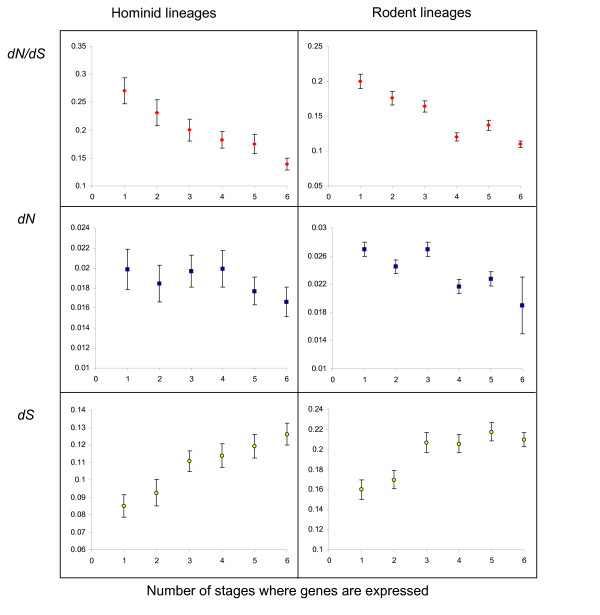
Substitution rates (*dN/dS*, *dN*, *dS*) and breadth of expression in hominid and rodent lineages.

We then analyzed the relationship between the breadth of expression and evolutionary rates. Genes expressed at only one parasitic stage (see methods for details) were characterized as unique and those expressed at all stages were characterized as ubiquitous. For both the hominid and rodent lineages, we observed a significant relationship between the breadth of expression and the rate of non-synonymous substitutions. On average, stage specific proteins (expressed at only one stage) evolve at a higher rate relative to ubiquitous ones (expressed at all stages) (Fig. 4 and Table 3). In contrast, synonymous variation shows a very different trend regarding the breadth of expression. The rate of synonymous substitutions increases with the breadth of expression: proteins expressed in a larger number of stages evolve at higher rates at synonymous sites. The obvious corollary of these observations is a negative relationship between the breadth of expression and *dN/dS *as shown in Figure 4. Note that we obtained similar relationships for rodents using data on the expression of *P. berghei *[5] except that we did not find any significant relationship between *dS *and the breadth of expression (data not shown).

**Table 3:** Relationship between gene expression, GC content and substitutions rates in both hominid's and rodent's *Plasmodium *parasites

	Hominid lineage	Rodent lineage
Expression-*dN/dS*	Rho = -0.26; *p *= 0	Rho = -0.26; *p *= 0
Expression-*dN*	Rho = -0.13; *p *= 0.008	Rho = -0.15; *p *= 0
Expression-*dS*	Rho = 0.18; *p *= 0	Rho = 0.113; *p *= 0.00001
Expression-GC1	Rho = 0.28; *p *= 0	Rho = 0.31; *p *= 0
Expression-GC2	Rho = 0.37; *p *= 0	Rho = 0.35; *p *= 0
GC1-*dN/dS*	Rho = -0.37; *p *= 0	Rho = -0.38; *p *= 0
GC2-*dN/dS*	Rho = -0.43; *p *= 0	Rho = -0.38; *p *= 0

A spearman rank test was used to analyze the correlation between variables (Rho: spearman correlation coefficient; *p*: *p-*value).

Our results are congruent with previous studies reporting relationships between breadth of expression and the rate of gene evolution in other organisms [14,15]. In both hominid and rodent lineages, highly expressed genes are generally more constrained than less expressed genes [16]. This observation is often attributed to the fact that proteins that are expressed in more diverse cellular environments are subjected to stronger functional constraints [14,15,17]. These results are consistent with the observation in both hominid and rodent parasites of a positive relationship between GC content at position 1 and 2 of codons (GC1-2) and the level of expression as well as a negative relationship between GC1-2 and *dN/dS *(Table 3). Highly and universally expressed genes are more GC-rich than lowly expressed genes which might thus reflect a codon bias, in particular for GC-rich codons. In other words, amino acids encoded by GC-rich residues are preferred and conserved in protein coding genes of the genus *Plasmodium*.

The positive relationship observed between the rate of synonymous substitutions and expression can potentially be explained by translational selection acting on synonymous codon sites of highly expressed genes [16]. Alternatively, an increase in transcription may simply increase the level of spontaneous mutations as demonstrated in certain bacteria [18].

## Conclusion

Our knowledge about the evolution of parasites responsible for malaria is increasing rapidly thanks to the availability of several completely sequenced genomes from species belonging to different lineages. As shown in the present study, different questions can be directly answered by comparing genomes from multiple *Plasmodium *species. Our comparative analysis suggests that, while there are a few aspects that are distinct among lineages, patterns of molecular evolution in the hominid parasite lineage are generally consistent with those observed in the rodent parasite lineage. In the murid lineage the most rapidly evolving genes are those involved in host-parasite interactions and those that are the least expressed. However, the evolution of the hominid lineage appears to be less constrained likely reflecting their historical lower effective size and an evolution driven by slightly deleterious mutations.

While we tried to be as exhaustive as possible in our comparison of the evolution of the genome of both species, this study is still imperfect and incomplete. A definitive study will require the use of a high-quality complete sequence for *P. reichenowi *as well as more population data on the rodent lineages. Analyses of polymorphisms in natural populations of both hominid and rodent lineages (in the rodent lineage this information is specifically lacking) is critical to better understand the nature and intensity of selection acting on different categories of genes.

Because for parasites with complex life-cycles (like *Plasmodium sp*.), the vector and the vertebrate host constitute very different environment, an interesting analysis would be to study the evolution of the genes exclusively expressed in the stages infecting the mammal host versus those only expressed in the stages infecting the mosquito vector. Such an analysis would however require the collection of more detailed expression profiles in both lineages.

## Methods

### Data sequences and alignments

For rodent malaria species, protein and nucleotide sequences for annotated genes for *P. berghei, P. chabaudi *and *P. yoelii yoelii *were obtained from The Plasmodium Genome Resource Database (Plasmodb [19]). Orthologous genes between the three species were obtained with BlastN using the criterion of best hits with scores of E < 1*10^-15 ^and at least 70% similarity in length. Only the groups of genes for which only one gene of each species corresponded to these criteria were conserved. All groups of coding sequences were aligned using Clustal W version 1.82 [20] (default parameters) using amino acid sequences followed by back-translation into nucleotides sequences using the original sequence provided by Plasmodb.

For hominid malaria species, protein and nucleotides sequences for annotated genes were obtained for *P. falciparum *only. For *P reichenowi*, Plasmodb provided only nucleotide contigs (release 09 July 2004) of a partial genome shotgun of approximately onefold coverage. The assembled contiguous sequences cover slightly less than one third of the *P. reichenowi *genome. Orthologous genes between the two species and their alignment were obtained following several steps. First, *P. falciparum *and *P. reichenowi *orthologues were obtained using BlastN with scores of E < 1*10^-15 ^and at least 70% similarity in length. Only groups of genes where we obtained only one gene of each species were kept. These groups were then aligned using ClustalW (V. 1.82) using default parameters. All the alignments were then very carefully checked by eye and corrected when necessary. Introns were deleted.

### Synonymous and non-synonymous substitution rate analyses

For both the rodent and hominid malaria gene groups, maximum likelihood estimates of rates of non-synonymous (*dN*) and synonymous (*dS*) substitutions, averaged over all branches, were obtained using PAML version 3.14 [21]. We used a codon-based model of sequence evolution with *dN *and *dS *considered as free parameters and the average nucleotide frequencies estimated from the data at each codon position (F3 × 4 MG model). The transition/tranversion bias *K *was estimated for each group of orthologous sequence. Because estimates of *dS *> 1 are more prone to error [22], only genes with *dS *≤ 1 were used for statistical calculations, yielding 843 and 3060 valid orthologues for hominid and rodents malaria groups respectively. For each gene, Likelihood Ratio Tests (LRT) were used to test whether the estimated *dN/dS *(ω) ratio differed significantly from 1 [23]. The tests were performed as bilateral tests of the hypothesis *H*_o_: *dN/dS *=1 versus the alternative hypothesis *H*_1_: *dN/dS ≠ 1 *for each group of sequence. Twice the difference of the log likelihood estimated for each hypothesis was then compared to a χ^2 ^distribution with one degree of freedom (*df*).

### Substitution rates and genomic features

To learn more about both synonymous and non-synonymous substitution patterns and their possible causes, we analyzed the effect of several genomic features such as the GC content, the level and timing of expression of genes and the function of proteins.

The biological process of the annotated proteins of *P. falciparum *was determined using GO (Gene ontology) annotations. A biological process is a series of events accomplished by one or more ordered assemblies of molecular functions. Examples of broad biological process terms are cellular physiological process or signal transduction. Classification of proteins was made using the software GENERIC GENE ONTOLOGY (GO) TERM MAPPER [24]. Because no such classification was available for any of the rodent species, *P. yoelii yoelii *genes were classified as their orthologous *P. falciparum *genes defined in The TIGR *Plasmodium yoelii yoelii *Genome Annotation Database.

For gene expression, we retrieved the mRNA abundance for genes of *P. falciparum *for different stages (rings, trophozoites, shizonts, merozoites, gametocytes and sporozoites) from [25]. For the rodent lineage, information on expression was available for *P. berghei *on a lower number of stages (asexual blood stages, gametocytes, ookinetes, oocysts and sporozoites) [5]. Such data precluded any possible rigorous comparison between the two lineages of parasites because of a lack of overlap between stages analyzed in the hominid and rodent lineages. We therefore decided to determine the timing of expression of rodent genes by using their orthology with *P. falciparum*, simply considering orthologous genes to be expressed at similar stages. We computed the breadth of expression for each gene in each lineage as the total number of different stages in which a gene is expressed.

GC content was computed using CODONW [26]. GC content quantifies the proportion of GC inside the gene.

## Authors' contributions

FP: conceived of the study, designed it, performed the sequence alignment and all the statistical analyses, wrote the manuscript. KMcG and JK.: participated in the sequence alignment and the analyses. PA: conceived of the study, and participated in its design and coordination and helped to draft the manuscript. All authors read and approved the final manuscript.

## Supplementary Material

Additional file 1 *P. falciparum *genes analyzed in the study and rates of evolution (*dS*, *dN*, *dN/dS*). Click here for file

Additional file 2 Orthologous genes of rodent Plasmodium species and their rates of evolution (*dS*, *dN*, *dN/dS*). Click here for file
